# Off- to in-season body composition adaptations in elite male and female endurance and power event athletics competitors: an observational study

**DOI:** 10.1186/s13102-024-00877-7

**Published:** 2024-04-22

**Authors:** Stefan Pettersson, Anton Kalén, Mikael Gustafsson, Stefan Grau, Andreas Caspers

**Affiliations:** 1 Center for Health and Performance, Department of Food and Nutrition, and Sport Science, University of Gothenburg, Gothenburg, Sweden; 2 Swedish Olympic Committee, Sofiatornet, Olympiastadion, Stockholm, Sweden; 3 Department of Computer Science, Electrical and Space Engineering, Luleå University of Technology, Luleå, Sweden; 4 Department of Sports Medicine, University Clinic Tübingen, Tübingen, Germany

**Keywords:** Dual-energy X-ray absorptiometry, BMC, BMD, Lean mass, Fat mass, Least significant change, Track and field

## Abstract

**Background:**

Monitoring elite athletes’ body composition (BC) is vital for health and optimizing performance in sports emphasizing leanness, such as athletics. This study aims to investigate and compare sex- and event-specific off-to in-season BC changes in endurance and power event athletics competitors.

**Methods:**

Elite male and female endurance athletes (> 800 m runners; *n* = 21) and power event athletes (sprinters, jumpers; *n* = 32) underwent dual-energy X-ray absorptiometry (DXA) scans for whole and regional lean mass (LM), fat mass (FM), bone mineral content (BMC), and density (BMD) during off-season (September-October) and in-season (April-May). Linear mixed models tested between-group off-season differences in BC, within-group off-season to in-season changes, and between-group differences in change. To assess meaningful or least significant changes (LSC) in BC, DXA precision errors were determined from two consecutive total body scans in a subsample of athletes (*n* = 30).

**Results:**

Male athletes (*n* = 26) gained significantly (*p* < 0.05) more body mass (BM; mean difference 1.5 [95% confidence interval (CI):0.5–2.4] kg), LM (843 [95% CI:-253:1459] g), and trunk LM (756 [-502:1156] g) than female athletes (*n* = 27). The proportion of changes in athlete’s BC exceeding the LSC threshold for LM and trunk LM were 70% and 65% in males, and 48% and 26% in females. Significant (*p* < 0.05) within-group off-season to in-season increases in LM were found for male endurance and power athletes, and female power athletes. All groups significantly increased BMD (*p* < 0.05). Only male and female power athletes had significant in- to-off-season increases in BMC. 80% of all athletes who had a meaningful increase in BMC belonged to the power event group. No significant within- or between group change in FM was observed.

**Conclusions:**

The present study found that male athletes gained more BM, LM and trunk LM than females. Within-group increases in regional and whole-body LM and BMC were predominantly found among power event competitors. Incorporating individual meaningful changes alongside traditional statistics provided additional insights into sex and event-group differences. Future research on elite athletic event groups should include DXA measurements closer to major outdoor-season competitions, coupled with site-specific measures (ultrasound, MRI) for better detection of subtle changes in LM and FM.

**Supplementary Information:**

The online version contains supplementary material available at 10.1186/s13102-024-00877-7.

## Introduction

Estimation of whole and regional body composition (BC) may be valuable for improving performance, injury prevention, and assessing health risks in athletes [1]. Generally, a high power-to weight-ratio, characterized by a greater proportion of lean mass (LM), of which a significant fraction is skeletal muscle, and a lower proportion of fat mass (FM) is considered crucial for locomotion and athletic performance [2]. On the other hand, an excessive focus on obtaining a low body mass (BM) and FM content has been related to negative outcomes, including decreased bone mineral density (BMD), reduced LM, disordered eating habits and menstrual dysfunction [3]. Monitoring changes of BC during an athletic season can provide vital information for coaches, athletes, and sport medicine professionals. It facilitates the evaluation of training program effectiveness, and identification of potential health and injury risks in sports emphasizing leanness such as athletics (track and field).

Numerous studies have examined BC in athletics athletes across various events, including middle- to long-distance running (800 m to marathon), sprinting (60–400 m including hurdles), jumping (long jump, triple jump, high jump, pole vault) and throwing (shot put, javelin, discus, hammer) [4–10]. Each event category imposes distinct physical demands that necessitate specific anthropometric dimensions and BC for optimal performance. For example, research on middle- to long-distance runners has demonstrated an inverse relationship between regional, total, and relative FM (%FM) and performance, while LM has been positively associated with higher aerobic capacity (e.g., V̇O_2_ max) in both male and female runners [11–14].

Similarly, sprinters and jumpers, known as power event athletes, typically exhibit a lean physique [15, 16]. Cross-sectional studies have demonstrated that the fastest sprinters tend to possess the greatest lower body muscle mass [17–19]. These findings suggest that during the transition from the off-season to the competitive season, endurance event athletes may benefit from a gradual reduction in total BM by decreasing FM while minimizing changes in LM. Conversely, power event athletes may benefit from increasing LM, particularly in thigh skeletal muscle mass thickness. Studies conducted by Stanforth et al. [9] and Carbuhn et al. [5] have reported reductions in FM and %FM, accompanied by increases in LM and BMD over the course of a competitive season in female sprinters and jumpers. However, it is essential to consider the disparities in training methods between sprinters/jumpers (involving sprinting, plyometric exercises, and power lifts) and middle- to long-distance runners (primarily comprising high-volume, low-intensity aerobic exercise). These divergent training approaches likely influence seasonal changes in BC in an event-specific manner [20, 21], however event comparisons have not been systematically researched with regards to BC methodology.

Dual-energy X-ray absorptiometry (DXA) has become an increasingly popular method for quantifying whole and regional FM, LM, BMD, and bone mineral content (BMC) in athletes [22]. While DXA is renowned for its excellent reproducibility, it is important to acknowledge that both biological factors (such as age, sex, body size, prior exercise, and acute food and fluid intake) and technical factors (DXA model, scan mode, and subject positioning on the scanning bed) can contribute to measurement errors [22]. Although adhering to a meticulous scanning protocol can mitigate some of these issues, understanding the measurement precision of the DXA device is crucial for interpreting meaningful changes when conducting consecutive BC measurements. The International Society for Clinical Densitometry (ISCD) recommends the use of the least significant change (LSC), which is calculated based on the precision error for a specific group. The LSC represents the smallest change in a BC variable that can be considered beyond the margin of error or the inherent variability in the measurement process. The objective of this study was to assess variations in BC changes between sex and event categories, and to determine the proportion of athletes who had BC changes that exceeded the DXA’s LSC.

## Methods

From a prospective cohort study consisting of 96 participants, which aimed to investigate risk factors and injuries over an eleven-month athletics season [23], a subsample of 53 elite athletics competitors (26 males (M), 27 females (F)) aged 22.2 ± 2.8 years, were selected for inclusion in this study. The majority of these athletes were part of the Swedish national team and regularly participated in international events. All participants were required to be over 18 years of age, resulting in the exclusion of *n* = 35 athletes from the initial cohort study. Additionally, one athlete who ceased training due to severe injury for more than three consecutive weeks (10% of the study duration), between the off-season and in-season DXA measurement periods, was excluded from the analysis. Four athletes underwent the off-season DXA measurement, but not the in-season scan and were therefore excluded. Three throwing event athletes were also excluded due to their significantly different training plans, both in terms of frequency and content, compared to the other included event categories (see below). The study was conducted in accordance with the declaration of Helsinki [24], and all procedures were approved by the Regional Ethical Committee in Gothenburg (dnr. 723–16), Sweden. Additionally, all athletes gave their written informed consent.

### DXA measurements

After an overnight fast, the athletes had their BM measured on a digital scale (Seca 764, Hamburg, Germany) to the nearest 0.1 kg while wearing underwear only. Height was measured to the nearest 0.5 cm with a standard wall-mounted stadiometer. Subsequently, BC was assessed using a fan-beam DXA scanner (iDXA GE Medical Systems, Madison, WI, USA) in the standard mode as determined automatically by the DXA software (EnCore, version 16.10) based on BMI. The athletes were positioned in a supine position, centrally aligned with their extremities fitting inside the measuring parameter and their feet and hands placed in custom-made radio-opaque positioning aids [22]. All scans were inspected for regions of interest misalignments and artefacts by two experienced technicians. The EnCore software automatically calculated whole- and regional FM, LM, BMC, BMD, and BMD Z-score. BMD Z-scores were calculated using the combined NHANES/Lunar database. Fat mass index (FMI) and fat-free mass index (FFMI) were calculated using the following formulas:

$${\rm{FMI}}({\rm{kg}}/{{\rm{m}}^2}) = {{{\rm{FM}} + {\rm{BMC}}\left( {{\rm{kg}}} \right)} \over {{\rm{Heigh}}{{\rm{t}}^2}\left( {{{\rm{m}}^2}} \right)}}$$

$${\rm{FFMI}}({\rm{kg}}/{{\rm{m}}^2}) = {{{\rm{LM}} + {\rm{BMC}}\left( {{\rm{kg}}} \right)} \over {{\rm{Heigh}}{{\rm{t}}^2}\left( {{{\rm{m}}^2}} \right)}}$$

The DXA assessments were conducted at two seasonal periods defined in this study: off-season assessments were completed after the outdoor athletic season (September-October), while in-season assessments were conducted at the beginning of the outdoor athletic season (April-May). DXA measurements were conducted during two seasonal periods: off-season assessments occurred after the outdoor athletic season (September-October), while in-season assessments were done at the beginning of the outdoor athletic season (April-May). Time between the off-season and in-season measurements was 5.7 ± 0.7 months. The athletes were classified into one of two event categories: *Endurance athletes* (M/F = 9/12) competing in running events from 800 m to 10,000 m and *Power athletes* (M/F = 17/15) including sprinters (M/F = 14/7), long- (F = 1), triple- (M/F = 2/1) and high jumpers (F = 1) and pole-vaulters (M/F = 1/5).

Following the ISCD guidelines [25], a precision estimation of the DXA equipment was performed on a subsample of the athletes (*n* = 30; M/F = 15/15; height 176 ± 6.9, BMI = 21.6 ± 2.0) who underwent duplicate scans with dismounting and repositioning on the scanning bed between measurements. The LSC was calculated following the recommended approach by the ISCD: RMS-SD × 2.77 (95% confidence interval (CI)), see Table 1.

**Table 1:** Reliability statistics for DXA estimated body composition measures in elite power (*n* = 15, 7 males, 8 females) and endurance athletes (*n* = 15, 8 males, 7 females) scanned twice with repositioning

		Precision			LSC (95% CI)			
Region	Variables	RMS-SD	CV (%)		RMS-SD	CV (%)		
Whole body	Lean mass (g)	179	0.3		496	0.9		
	Fat mass (g)	143	1.3		396	3.7		
	Fat mass (%)	0.2	1.4		1	3.8		
	BMC (g)	8	0.3		21	0.7		
	BMD (g/cm^2^)	0.006	0.4		0.016	1.2		
Arms	Lean mass (g)	67	1.2		186	3.3		
	Fat mass (g)	35	3.7		96	10.3		
	BMC (g)	4	1.0		11	2.6		
Trunk	Lean mass (g)	201	0.8		557	2.2		
	Fat mass (g)	113	2.5		312	7		
	BMC (g)	7	0.8		20	2.3		
Legs	Lean mass (g)	151	0.8		417	2.1		
	Fat mass (g)	73	2.2		202	6.0		
	BMC (g)	5	0.4		14	1.1		

*Note* BMC; bone mineral content, BMD; bone mineral density, CI; Confidence interval, CV; Coefficient of variation, LSC; least significant change, RMS-SD; root mean squared standard deviation.

### Training frequencies

Supplementary Fig. 1 provides information on the monthly number of training sessions and training days from October to April. The mean training hours per week (14 ± 3 h) did not significantly differ between event categories or sexes. Throughout the study period, the endurance athletes performed approximately two resistance training sessions per week, while the majority of the power athletes engaged in four strength sessions per week from October to December and two sessions per week from January to April, (as per personal communication with the strength and conditioning coaches).

### Statistical analysis

Linear mixed model was fitted for each variable using cluster-robust standard errors. Time (off-season or in-season), sex (male or female), and event type (endurance or power), along with their two- and three-way interactions were included as fixed factors. The model contained a random intercept for each athlete. Difference in estimated marginal means from the models were used to test for between-group off-season differences in BC, within-group off-season to in-season change, and for between-group differences in change.

Based on the LSC calculations (Table 1), the off-season to in-season change of each athlete was categorized as meaningful increase, no change or decrease. All statistical analyses were conducted using R software (version 4.1.2). A significance level of *p* < 0.05 was used to determine statistical significance, and the Benjamini–Hochberg procedure was applied to control for the false discovery rate in all contrasts.

## Results

### Off-season sex- and event-specific differences in BC

The overall off- and in-season whole and regional BC estimates of the athletes are presented in Table 2, and off-season sex and event differences (mean [lower: upper, 95% CI]) can be found in Supplementary Table 1. Male athletes had significantly higher BM, FFMI, BMD, BMD Z-score and total and regional LM and BMC than female athletes *(p* ≤ 0.042). Males, in comparison to females, also had lower FMI, %FM and total and regional FM (*p* ≤ 0.006; Table 2 and Supplementary Table 1), except for trunk FM (mean difference − 568; [-1280: 144] g; *p* = 0.116).

Off-season event comparisons showed that power athletes (*n* = 32) had significantly higher BM, FFMI, BMD, BMD Z-score, FMI, and FM, and total- and regional LM and BMC (*p* ≤ 0.02) than endurance athletes (*n* = 21), while no significant differences between events in % FM and arms FM were observed.

Male power athletes had significantly higher FFMI, FMI, BMC, BMD and BMD Z-score (*p* ≤ 0.046) than male endurance athletes, but no significant differences in LM, FM, %FM or BM were observed (mean group BM difference 6.0 [0.6: 11.4] kg; *p* = 0.051). Female power athletes had, except for total %FM (*p* = 0.058) and arms FM (*p* = 0.072), significantly higher off-season BMD, BMD Z-score and total- and regional LM, BMC, and FM than the female endurance athletes (*p* ≤ 0.041, see Table 2 and Supplementary Table 1).

### Sex and event-type off- to in-season BC changes

**Fig. 1:**
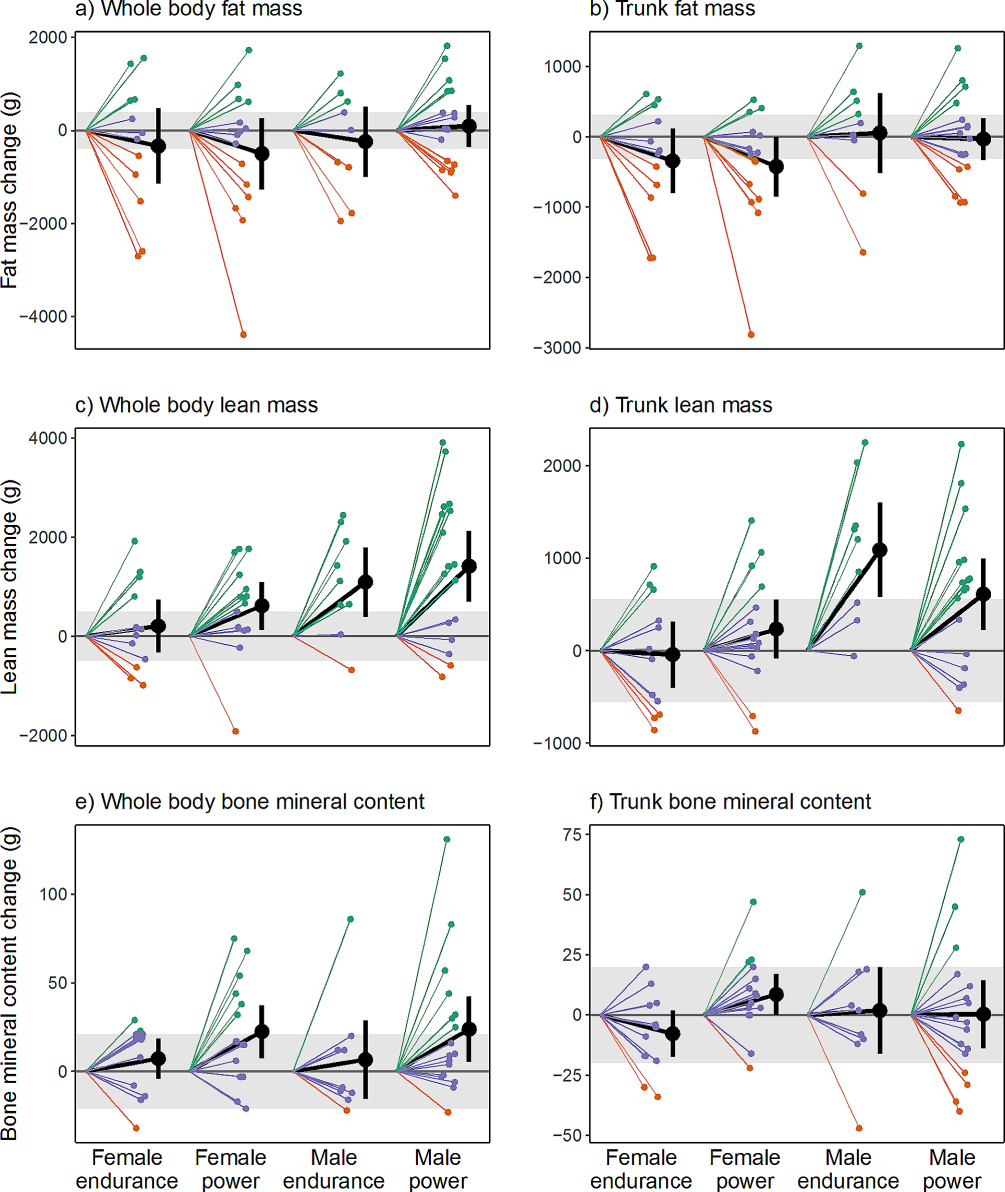
Individual off-season to in-season changes in whole body (a, c, e) and trunk (b, d, f) composition in elite male and female endurance and power athletics event competitors (*n* = 53). Grey area represents least significant change (LSC)-95% confidence interval, i.e., precision error of DXA measurement. The green lines represent athletes who experienced off- to in-season changes in LM, FM, and BMC that exceeded the LSC threshold. Red lines indicate athletes who experienced reductions surpassing the LSC threshold, while purple lines represent athletes who neither increased nor reduced beyond the LSC threshold

**Fig. 2:**
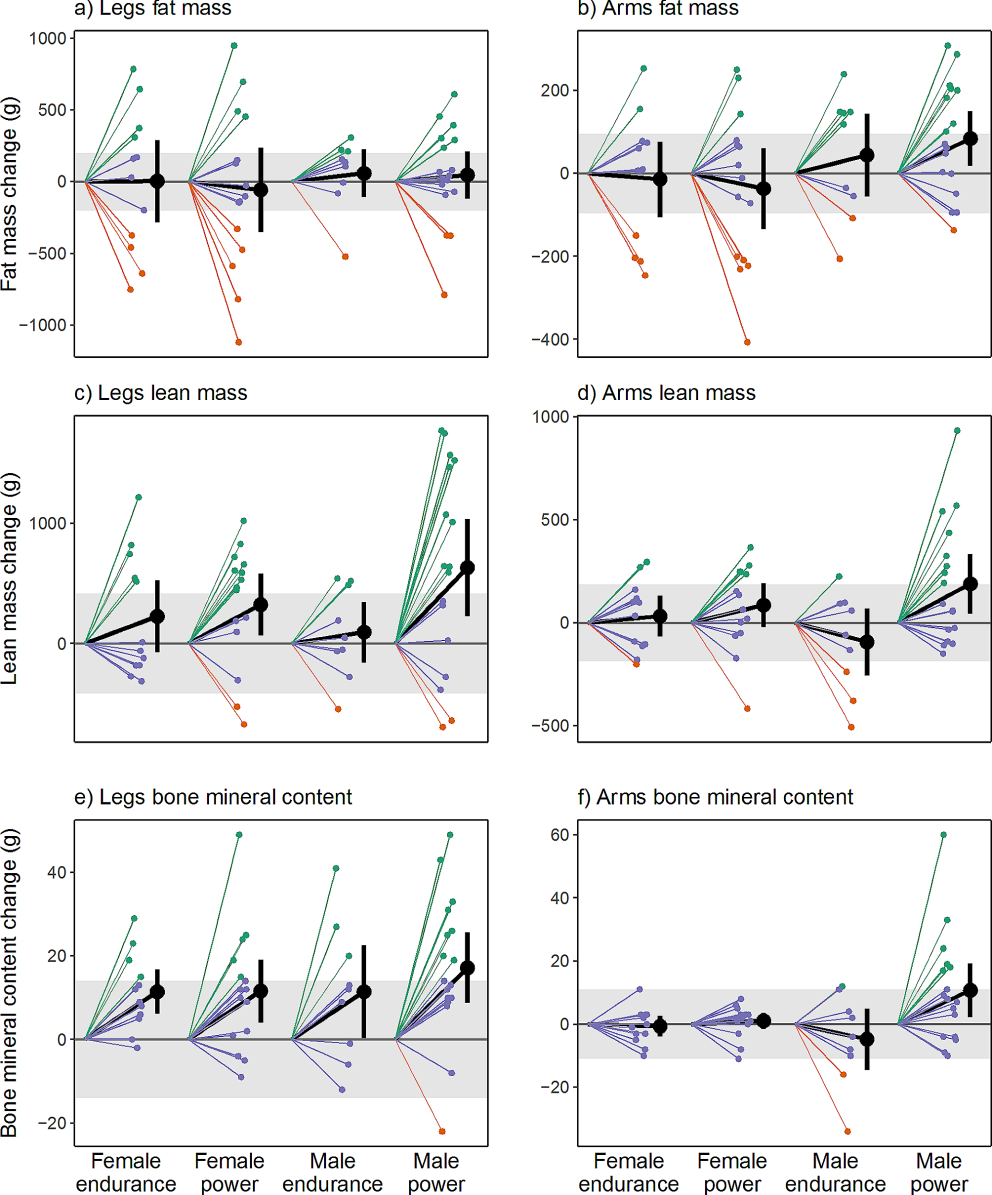
Individual off-season to in-season changes in legs (a, c, e) and arms (b, d, f) composition in elite male and female endurance and power athletics event competitors (*n* = 53). Grey area represents least significant change (LSC)-95% confidence interval, i.e., precision error of DXA measurement. The green lines represent athletes who experienced off- to in-season changes in LM, FM, and BMC that exceeded the LSC threshold. Red lines indicate athletes who experienced reductions surpassing the LSC threshold, while purple lines represent athletes who neither increased nor reduced beyond the LSC threshold

Table 2 presents the within-group differences in total and regional body composition estimates from the off-season to the in-season. Additionally, Fig. 1 illustrates the changes in total body and trunk LM, FM, and BMC from the off- to in-season periods, while Fig. 2 displays the alterations in BC specifically in the extremities (arms and legs). No significant change in absolute or %FM was observed. The male athletes gained 1.1 kg in BM (0.5: 1.8 kg; *p* = 0.004), a BM change which mainly consisted of LM accretion (mean change 1257 g, [757: 1757] g, *p* < 0.001) in the trunk and legs (mean change 851 g and 363 g, *p* = < 0.001 and *p* = 0.009, respectively). Consequently, mean FFMI increased by 0.4 kg/m^2^ (*p* < 0.001) and significant increases in BMD, BMD Z-score, total BMC, and legs BMC were also noted (*p* ≤ 0.015).

**Table 2:** Elite athletics competitors (*n* = 53) anthropometric and body composition (BC) characteristics (mean ± SD) at off-season to in-season including absolute off- to in-season BC change

	Female athletes (*n* = 27)								Male athletes (*n* = 26)								All athletes (*n* = 53)						
	Endurance (*n* = 12)				Power (*n* = 15)				Endurance (*n* = 9)				Power (*n* = 17)				Females (*n* = 27)				Males (*n* = 26)		
	Off-season	In-season	Change		Off-season	In-season	Change		Off-season	In-season	Change		Off-season	In-season	Change		Off-season	In-season	Change		Off-season	In-season	Change
Age (yrs)	23.0 ± 3.8	˗	˗		22.4 ± 2.8	˗	˗		20.9 ± 1.8	˗	˗		22.3 ± 2.3	˗	˗		22.6 ± 3.3	-	˗		21.8 ± 2.2	-	˗
Height (m)	1.7 ± 6.2	˗	˗		1.71 ± 2.7	˗	˗		1.84 ± 5.4	˗	˗		1.84 ± 5.3	˗	˗		1.71 ± 4.4	-	˗		1.84 ± 5.0	-	˗
BM (kg)	56.6 ± 5.6†	55.8 ± 5.4	-0.8 ± 1.3		64.2 ± 5.8	64.3 ± 5.5	0.1 ± 2.1		69.3 ± 7.1	70.1 ± 6.4	0.8 ± 1.6		75.3 ± 5.2	76.8 ± 5.1	1.4 ± 1.7*		60.8 ± 6.8†	60.5 ± 6.8	-0.3 ± 1.8‡		73.2 ± 6.5	74.5 ± 6.3	1.2 ± 1.7*
FFMI (kg/m^2^)	16.2 ± 0.9†	16.3 ± 1.0	0.1 ± 0.3		17.4 ± 1.2	17.7 ± 1.2	0.2 ± 0.3*		18.1 ± 1.1†	18.4 ± 0.8	0.3 ± 0.3*		19.5 ± 1.4	20.0 ± 1.3	0.4 ± 0.4*		16.9 ± 1.2†	17.1 ± 1.3	0.2 ± 0.3		19.0 ± 1.5	19.4 ± 1.4	0.4 ± 0.4*
FMI (kg/m^2^)	3.3 ± 1†	3.2 ± 0.8	-0.1 ± 0.5		4.2 ± 1.0	4.0 ± 0.7	-0.2 ± 0.5		2.4 ± 0.4†	2.3 ± 0.4	-0.1 ± 0.3		2.8 ± 0.4	2.8 ± 0.4	0.0 ± 0.3		3.8 ± 1.1†	3.7 ± 0.9	-0.1 ± 0.5		2.6 ± 0.5	2.6 ± 0.5	0.0 ± 0.3
LM (kg)	44.3 ± 3.7†	44.6 ± 3.8	0.2 ± 0.9		48.8 ± 3.9	49.4 ± 4.0	0.6 ± 0.9*		58.2 ± 5.7	59.3 ± 4.9	1.1 ± 1.0*		62.4 ± 4.8	63.8 ± 4.9	1.4 ± 1.5*		46.8 ± 4.4†	47.2 ± 4.5	0.4 ± 0.9‡		60.9 ± 5.4	62.3 ± 0.3	1.3 ± 1.3*
FM (kg)	9.6 ± 3.3†	9.2 ± 2.6	-0.3 ± 1.4		12.5 ± 2.9	12.0 ± 2.1	-0.5 ± 1.5		8.1 ± 1.8	7.8 ± 1.8	-0.2 ± 1.1		9.4 ± 1.1	9.5 ± 1.2	0.1 ± 0.9		11.2 ± 3.4†	10.8 ± 2.7	-0.4 ± 1.4		8.9 ± 1.5	8.9 ± 1.6	0.0 ± 1.0
FM (%)	16.7 ± 4.6	16.4 ± 3.6	-0.3 ± 2.2		19.3 ± 3.3	18.5 ± 2.5	-0.8 ± 1.6		11.6 ± 2.1	11.1 ± 2.1	-0.5 ± 1.5		12.5 ± 1.6	12.4 ± 1.6	-0.1 ± 1.1		18.1 ± 4.1†	17.6 ± 3.2	-0.5 ± 1.9		12.2 ± 1.8	11.9 ± 1.8	-0.2 ± 1.3
BMC (g)	2539 ± 270†	2546 ± 273	7 ± 20		2931 ± 250	2953 ± 260	22 ± 29*		3158 ± 373†	3165 ± 376	7 ± 33		3588 ± 350	3612 ± 344	24 ± 38*		2757 ± 322†	2772 ± 332	16 ± 26*		3440 ± 408	3457 ± 410	18 ± 37
BMD (g/cm^2^)	1.26 ± 0.08†	1.28 ± 0.09	0.02 ± 0.04		1.38 ± 0.08	1.41 ± 0.09	0.04 ± 0.03*		1.29 ± 0.09†	1.32 ± 0.09	0.03 ± 0.04		1.45 ± 0.09	1.48 ± 0.09	0.04 ± 0.03*		1.32 ± 0.09†	1.35 ± 0.12	0.03 ± 0.04*		1.39 ± 0.12	1.42 ± 0.12	0.03 ± 0.03*
BMD Z-score	2.07 ± 0.75^†^	2.42 ± 0.73	0.35 ± 0.35*		3.1 ± 1.06^†^	3.54 ± 1.27	0.44 ± 0.46*		0.86 ± 0.69^†^	1.31 ± 0.90	0.46 ± 0.49*		2.35 ± 0.83^†^	2.79 ± 0.92	0.44 ± 0.28*		1.83 ± 1.06^†^	2.28 ± 1.15	0.45 ± 0.35*		2.62 ± 1.05^†^	3.02 ± 1.18	0.40 ± 0.41*
**Regions**																							
Trunk LM (kg)	21.8 ± 1.6	21.8 ± 1.6	0.0 ± 0.6		23.1 ± 1.8	23.4 ± 1.8	0.2 ± 0.6		27.5 ± 2.9	28.6 ± 2.4	1.1 ± 0.8*		29.3 ± 2.1	29.9 ± 2.5	0.6 ± 0.8*		22.6 ± 1.8†	22.7 ± 1.9	0.1 ± 0.6‡		28.7 ± 2.5	29.5 ± 2.5	0.8 ± 0.8*
Trunk FM (kg)	4.0 ± 1.5†	4.0 ± 1.2	-0.3 ± 0.8		5.2 ± 1.1	5.2 ± 1.1	-0.4 ± 0.8		2.7 ± 0.8†	2.7 ± 0.7	0.1 ± 0.9		3.2 ± 0.6	3.2 ± 0.6	0.0 ± 0.6		4.4 ± 1.7	4.0 ± 1.2	-0.4 ± 0.8		3.9 ± 0.9	3.9 ± 0.8	0.0 ± 0.7
Trunk BMC (g)	698 ± 105†	691 ± 100	-8 ± 17		879 ± 98	887 ± 96	9 ± 16		862 ± 131†	864 ± 133	2 ± 27		1083 ± 113	1084 ± 108	0 ± 29		798 ± 135†	780 ± 138	1 ± 18		1007 ± 158	1008 ± 156	1 ± 28
Arms LM (kg)	4.3 ± 0.5†	4.3 ± 0.5	0.0 ± 0.2		5.2 ± 0.6	5.3 ± 0.7	0.1 ± 0.2		6.7 ± 0.8†	6.6 ± 0.7	-0.1 ± 0.2		7.5 ± 0.8	7.7 ± 0.8	0.2 ± 0.3*		4.8 ± 0.8†	4.9 ± 0.8	0.1 ± 0.2		7.2 ± 0.9	7.3 ± 0.9	0.1 ± 0.3
Arms FM (kg)	1.2 ± 0.5	1.2 ± 0.4	0.0 ± 0.2		1.5 ± 0.3	1.4 ± 0.3	0.0 ± 0.2		1.0 ± 0.3	1.1 ± 0.2	0.0 ± 0.1		1.1 ± 0.2	1.2 ± 0.2	0.1 ± 0.1*		1.3 ± 0.4†	1.3 ± 0.4	0.0 ± 0.2		1.1 ± 0.2	1.1 ± 0.2	0.1 ± 0.1
Arms BMC (g)	309 ± 36†	308 ± 37	-1 ± 6		366 ± 53	367 ± 55	1 ± 5		428 ± 56†	423 ± 55	-5 ± 14		484 ± 62	495 ± 61	11 ± 17*		341 ± 54†	341 ± 56	0 ± 5		464 ± 65	470 ± 67	5 ± 18
Legs LM (kg)	15.4 ± 1.7†	15.6 ± 1.8	0.2 ± 0.5		17.4 ± 1.9	17.7 ± 1.8	0.3 ± 0.5*		20.6 ± 2.1	20.7 ± 1.9	0.1 ± 0.4		22.2 ± 2.3	22.8 ± 1.9	0.6 ± 0.8*		16.5 ± 2.1†	16.8 ± 2.1	0.3 ± 0.5*		21.7 ± 2.3	22.1 ± 2.1	0.4 ± 0.7*
Legs FM (kg)	4.0 ± 1.5†	4.0 ± 1.2	0.0 ± 0.5		5.2 ± 1.1	5.2 ± 1.1	-0.1 ± 0.6		2.7 ± 0.8	2.7 ± 729 0.0	0.1 ± 0.2		3.2 ± 0.6	3.2 ± 0.6	0.0 ± 0.3		4.7 ± 1.4†	4.6 ± 1.3	0.0 ± 0.5		3.0 ± 0.7	3.1 ± 0.7	0.1 ± 0.3
Legs BMC (g)	1019 ± 123†	1031 ± 125	11 ± 9*		1138 ± 96	1150 ± 105	12 ± 15*		1321 ± 153†	1333 ± 163	11 ± 17		1475 ± 150	1493 ± 152	17 ± 17*		1085 ± 123†	1097 ± 127	12 ± 12*		1422 ± 166	1437 ± 171	15 ± 17*

*Note* BM, body mass; LM, lean mass; FM, fat mass; FM (%) relative fat mass; BMC, bone mineral content; BMD; bone mineral density, FFMI, fat free mass index; FMI, fat mass index. Data are presented as mean ± SD. *Indicates significant (p < 0.05) within-group changes in BC variables, ^†^*indicates significant between-group differences in off-season BC variables (between event types within each sex and between sex in all athletes), and*^‡^*indicate between-group differences in change in BC variables (between event types within each sex and between sex in all athletes)*

**Fig. 3:**
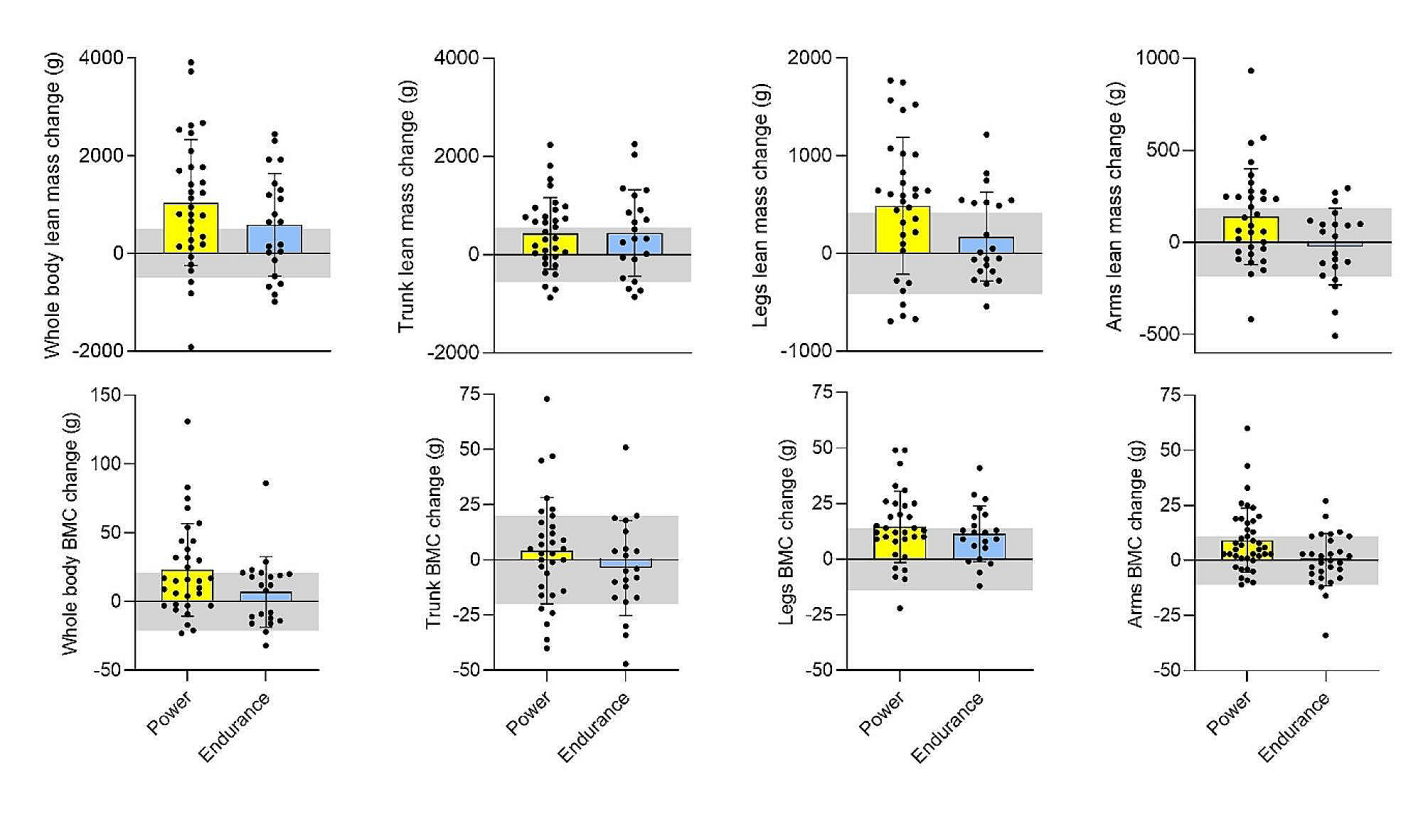
Mean off- to in-season whole and regional lean mass and bone mineral content (BMC) changes in elite male and female power (*n* = 32) and endurance (*n* = 21) athletics event competitors. Grey area represents least significant change (LSC)-95% confidence interval, i.e., precision error of DXA measurement

**Fig. 4:**
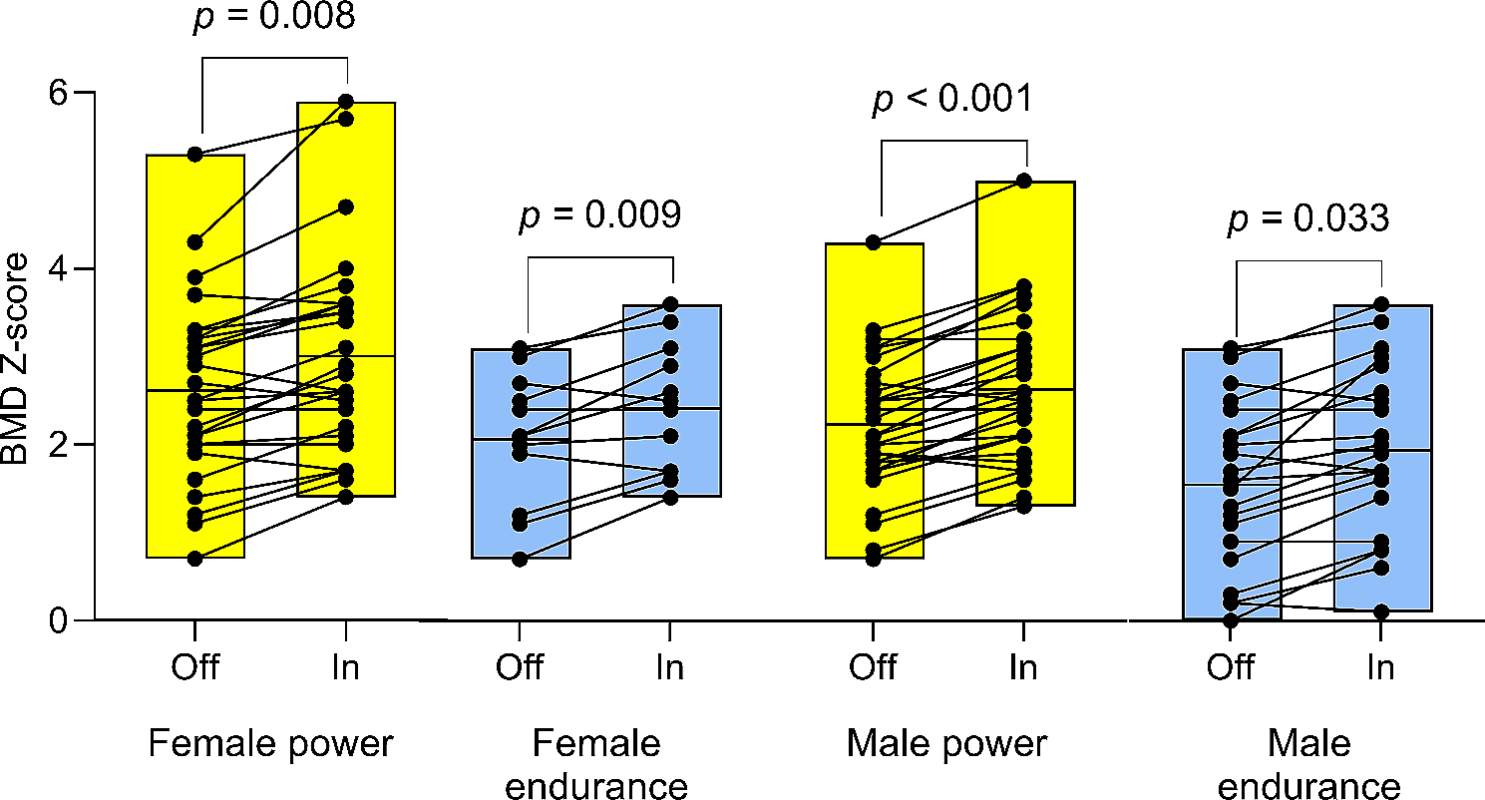
Off-season (Off) to in-season (In) changes in whole-body bone mineral density (BMD) Z-scores among elite female and male competitors in power (F: *n* = 15, M: *n* = 17) and endurance (F: *n* = 12, M: *n* = 9) athletics events. The box plots depict the mean BMD Z-score and the range of values (min-max distribution). Significant indicators denote within-group off- to in-season changes

Figure 3 illustrates mean changes in total and regional LM and BMC for power and endurance athletes, respectively. The male power athletes significantly increased BM (mean change 1.4 [0.6: 2.3] kg), LM (1416 [703 to 2130] g) and arms, legs, and trunk LM and FFMI (*p* ≤ 0.023). BMD, BMD Z-score (Fig. 4), total BMC, and legs and arms BMC were also higher in-season than at off-season (*p* ≤ 0.023), while no significant change was noted for FM variables except an increase in arms FM (*p* = 0.023). In male endurance athletes, increments in LM (1097 [396: 1797] g; *p* = 0.019), trunk LM (1089 g; *p* = 0.001) and FFMI (0.3 kg/m^2^; *p* = 0.019) were the only significant BC changes noted.

For the female athletes (*n* = 27) no significant changes were found for whole or regional FM and LM variables, except legs LM which increased by 275 g from off-season to in-season (79: 475 g, *p* = 0.030). Furthermore, female athletes BMD, BMD Z-score, total BMC and legs BMC were significantly (*p* ≤ 0.030) higher at in-season than off-season.

Female power athletes significantly increased LM (mean change 618 [133: 1103] g, *p* = 0.041) and FFMI (0.2 kg/m^2^, *p* = 0.041), total BMC and BMD (22 g and 0.039 g/cm^2^, *p* = 0.024 and *p* = 0.001, respectively) and BMD Z-score (*p* = 0.008) but also legs LM (324 [67: 581] g; *p* = 0.041) and legs BMC (12 [4: 19] g; *p* = 0.024). In the female endurance athlete group, the only significant BC change observed over the course of the season were increased legs BMC (mean change 11 [6: 17] g; *p* = 0.001) and BMD Z-score (mean change 0.35 [0.15:0.55] *p* = 0.009).

### Sex and event-type differences in off- to in-season BC changes

The results of the sex and event-type differences in off- to in-season BC changes, including 95% CI and p-values, are presented in Supplementary Table 2. Male athletes gained significantly more BM (mean difference 1.5 [0.5: 2.4] kg; *p* = 0.027), LM (mean difference 843 [-253: 1459] g; *p* = 0.048) and Trunk LM (mean difference 756 [-502: 1156] g; *p* = 0.007) than female athletes. No other significant differences in BC change were found for sex and event-type.

### Meaningful off- to-in-season BC changes

The figures (Figs. 1, 2 and 3) present the number of athletes surpassing the LSC estimates for alterations in whole- and regional LM, BMC, BMD, and FM between the off-season and in-season, while additional statistical inferences are provided in Supplementary Table 3. Notably, 58% of all athletes (20 power and 11 endurance) experienced a substantial increase (> 496 g) in LM whereas six athletes (11% of the total sample) exhibited a meaningful decrease in LM (> -496 g). In terms of regional LM changes, 51% and 45% respectively demonstrated a significant increase in leg LM (> 417 g) and trunk LM (> 417 g), while five athletes exhibited a decrease in leg LM (see Figs. 1d and 2c).

30% of athletes exhibited a verifiable increase, while 40% demonstrated a noteworthy decrease in whole-body FM (LSC = ± 396 g). Among all athletes, 32% (*n* = 17) exhibited an increase in leg FM (> 202 g) with a similar relative distribution across sex and event groups (27–35%, Supplementary Table 4). One-third of participants (*n* = 13) displayed a meaningful decrease in leg FM (Supplementary Table 4 and Fig. 2a).

All participants had high off-season BMD (1.35 ± 0.11 cm^2^, min-max = 1.14–1.65 cm^2^) and high BMD Z-score (2.23 ± 1.1, min-max = 0.0–5.3) values; however, 30% (*n* = 16) demonstrated a meaningful increase in whole-body BMC (> 21 g), with the majority (81%) comprising power athletes (*n* = 13; M/F = 8/5, Fig. 1e). Three athletes showcased a decrease in whole-body BMC (Figs. 2e and 3) that exceeded the LSC. 38% of all athletes experienced a meaningful increase (> 14 g) in leg BMC, with a larger proportion of the male power athletes (47%; *n* = 8) compared to the other three groups (33%, respectively).

## Discussion

The study aimed to investigate and compare changes in BC among elite athletics competitors during the transition from off-season to in-season, focusing on sex- and event-related variations. The results highlighted significant increases in BM, total LM, and trunk LM in male athletes compared to females. Significant within-group off-season to in-season increases in regional and whole-body LM, BMC and BMD Z-score were more prominent in power athletes than in endurance athletes. About 60% and 30% of all athletes demonstrated a meaningful increase in LM and BMC, respectively. For BMC, 80% of power athletes had a true increase, distributed equally between sexes. No significant changes were observed in any variable related to FM, except for an increase in arms FM in male power athletes.

### Off- to in-season changes in LM

Sex comparisons revealed that male athletes, in contrast to females, exhibited a significant increase in LM and trunk LM from the off-season to the in-season period. The initial lower LM in females during the off-season may induce bias in favor of their increases in both total and trunk LM [26]. Other factors, such as differences in FFMI during the off-season could also contribute to the observed variation in LM accrual. Although we did not find any significant within- or between-group associations between off-season LM/FFMI and change in LM/FFMI (likely due to small sample sizes), female athletes in power and endurance events exhibited higher off-season FFMI compared to normative sport-specific FFMI values [27]. In contrast, male athletes had lower off-season muscularity index (FFMI = 19.0 ± 1.5) compared to previously reported values in aged-matched male track and field athletes (FFMI = 22.4 ± 3.7) [28], suggesting a greater potential for LM increase than in the female athletes. However, it is important to note that skeletal muscle mass only makes up for approximately 30% of total trunk LM [29]. Still, 68% of the total LM accrual in male athletes consisted of trunk LM, while in females, this proportion was 23%. Previous research on healthy non-athletes [30] and world-class powerlifters [31] has indicated that females generally exhibit lower trunk muscle thickness/mass compared to males, implying a lesser potential for significant increases in trunk skeletal mass and thus absolute trunk LM. Sex-differences in trunk LM accrual was also observed regarding meaningful changes, with 25% of all female endurance and power athletes exceeding the LSC, compared to 65% of their male counterparts, respectively.

While no significant between-event-group comparisons were found, there were significant within-group increases in LM observed in all groups, except for female endurance athletes. The off- to in-season increases in LM in the male and female power event group concur with previous findings in male and female NCAA Division 1 jumpers and sprinters [5, 32] studied during the same period of the training year as the present study. Correspondingly, Trinschek and colleagues [33] showed that the percentage of LM in elite male polish endurance runners and sprinters significantly increased between consecutive phases of the annual training cycle. Considering that even modest resistance training frequencies have been shown to be effective in increasing muscle mass and strength [34] the inclusion of two (for endurance athletes) up to four (for power athletes) resistance training sessions per week during the study period is a plausible explanation as to why almost all athletes (87%) in the present study were able to maintain (28%) or increase (59%) LM above the LSC threshold. However, although the DXA in this study demonstrated commendable short-term precision error values for total LM (0.3%), below the ISCD [25] recommended limit (LM < 2%), future research should consider combining DXA with site-specific measures (ultrasound, CT, MRI) for improved detection of subtle seasonal changes in LM between different athletic events [35].

### Off- to in-season changes in FM

Contrarily to LM changes, no significant within- or between-group reductions in whole body FM, or %FM, were observed. Off-season %FM ranged from 11.6 to 19.3% (endurance males and female power athletes, respectively) which is similar to some studies [9, 33], but higher than other previously published DXA-derived off-season FM values in elite athletics athletes [32]. Low initial (off-season) FM levels of athletes have previously been suggested as a possible explanation for the lack of change observed during different seasons of training and/or competition [36]. However, considering that the athletes off-season FM levels in the present cohort are well above the minimum %FM cut-off values proposed by the International Olympic Committee´s Medical Commission [3] (i.e., < 5 and 12%, males and females respectively), an alternative explanation for the lack of significant off- to in-season change in FM could be the timing of the present study’s BC measurements in relation to the annual training cycle. A systematic review [37] demonstrated that male and female endurance athletes had a significantly lower percentage of FM during the competition phase compared to the preparation phase, and Mangine et al. [32] recently reported a ∼ 5% reduction in %FM in collegiate male and female sprint and power athletes between the return from winter break (early January) and the end of the indoor season (early April) but a substantially higher reduction (∼ 20%) between April and late May (before the NCAA Championships). Thus, future research on seasonal changes in FM should consider additional BC measurements closer to major outdoor-season competitive events. Nevertheless, the results of the present investigation are still highly relevant since prioritization of BM/FM loss in athletes is generally recommended to take place before, not during, the competitive season [3]. It is also worth noting that although the present study’s short-term precision error value for total FM (1.3%) was below the recommended limit by the ISCD [25] (< 3%), it was still considerably high compared to whole body CV’s of LM, BMC and BMD.

### Off- to in-season changes in BMC and BMD

Within-group analysis revealed a significant off- to in-season increase in BMD Z-score for all groups. However, only male and female power athletes demonstrated a significant increase in whole-body BMC. Carbuhn et al. [5] discovered similar off- to in-season mean increases in absolute and relative BMC (26 g and ∼ 0.9% BMC increase, respectively) among collegiate female sprinters and jumpers, as observed in our female power event cohort (22 g and ∼ 0.8% BMC increase, respectively). However, the present LSC-analysis revealed that only 40% of the female sprinters and jumpers experienced a meaningful increase in BMC (≥ 21 g). Furthermore, the majority (∼ 80%) of athletes with a meaningful increase in BMC belonged to the power event group, with equal relative distribution between sexes. However, within-group off- to in-season changes in BMD found that both male/female power athletes and female endurance athletes significantly increased BMD (male endurance athletes *p* = 0.054; BMD Z-score significantly increased in all groups). This finding aligns with previous research [38] indicating that while the axial loading and weight-bearing nature of middle- to long-distance running can enhance bone turnover and increase BMD, the heavier power event athletes, who impose a greater vertical load on the skeleton, may further augment BMD deposition. Furthermore, all athletes were found to have high whole-body BMD (e.g., a Z-score above − 1.0) [39]. It is also important to note that three athletes experienced a meaningful decrease in BMC, highlighting the clinical relevance of LSC estimates [3].

### Strength and limitations of the study

The study prioritized a high level of measurement precision by conducting fasted state DXA scans with a standardized positioning protocol [22]. Another strength of the study was the adherence to recommended guidelines by the ISCD in performing reliability statistics on a subsample of the athletes being studied. Limitations include the small sample size of the participant group, the lack of access to more detailed training diaries, and the absence of data on the athletes’ dietary intake. Training diaries should be developed in close cooperation with the athletes’ coaches to increase compliance, and could be based on e.g., volume and type of training. A 7-day diet-monitoring period during both off- and in-season could have offered insights into implementing periodized energy and macronutrient intakes for changes in BC [40]. Finally, previous DXA research has illustrated racial differences in body proportion, fat-free mass (FFM) density, and BMD [41]. Thus, the limited ethnic diversity observed within the current study’s cohort (all Caucasian/White) hinders the ability to generalize these findings to broader populations [41].

## Conclusions

The present study revealed significant sex differences in off-to-in-season changes in total BM, LM, and trunk LM. Significant within-group increases were observed in regional and whole-body LM, BMC, predominantly among power event athletes. Even though all athletes had high off-season BMD, almost all athletes increased BMD over the six-month period. Supplementing traditional statistics with individual meaningful changes in BC also yielded additional insights into the practical relevance of off-to-in-season differences. For example, even though there was a statistically significant difference in BMC in male and female power athletes from off-to-in-season, only 40% in each group showed a meaningful change. Furthermore, it is advisable to include additional DXA measurements closer to major outdoor-season events, coupled with site-specific measures (ultrasound, MRI), to enhance the detection of subtle changes in LM and FM.

### Electronic supplementary material

Below is the link to the electronic supplementary material.

Supplementary Material 1

## Data Availability

The datasets used during the current study are available from the corresponding author on reasonable request.

## Abbreviations

BC: Body composition
BM: Body mass
BMC: Bone mineral content
BMD: Bone mineral density
CI: Confidence interval
CV: Coefficients of variation
DXA: Dual-energy X-ray absorptiometry
FFMI: Fat-free mass index
FM: Fat mass
FMI: Fat mass index
ISCD: International Society for Clinical Densitometry
LSC: Least significant change
SD: Standard deviation
