# Supplementary material for: Off- to in-season body composition adaptations in elite male and female endurance and power event athletics competitors: an observational study
Source: BMC Sports Sci Med Rehabil. 2024 Apr 22;16:90. doi: 10.1186/s13102-024-00877-7 (PMC11034045; doi:10.1186/s13102-024-00877-7)
Supplement: Supplementary file 1 — Supplementary Material 1 [file 13102_2024_877_MOESM1_ESM.docx]

Supplementary Figure 1. Monthly number of training sessions (A) and (B) training days for endurance (n=21) and power event athletics competitors (n=32).

**A**

**B**

Supplementary Table 1. Between-group off-season differences in athletics athletes regional and whole body composition variables (n=53; females=27, males=26).

|  |  | | |  | | | Female | | | Male | | |
| --- | --- | --- | --- | --- | --- | --- | --- | --- | --- | --- | --- | --- |
|  | Male − Female | | | Power − Endurance | | | Power − Endurance | | | Power − Endurance | | |
| Variable | Diff | 95% CI | p-val | Diff | 95% CI | p-val | Diff | 95% CI | p-val | Diff | 95% CI | p-val |
| BM (kg) | 11.9 | 8.4, 15.4 | **< 0.001** | 6.8 | 3.3, 10.3 | **0.001** | 7.5 | 3.1, 11.9 | **0.005** | 6.0 | 0.6, 11.4 | 0.051 |
| FFMI (kg/m^2^) | 2.0 | 1.3, 2.6 | **< 0.001** | 1.3 | 0.7, 2.0 | **0.001** | 1.2 | 0.4, 2.0 | **0.011** | 1.4 | 0.4, 2.4 | **0.026** |
| FMI (kg/m^2^) | -1.2 | -1.6, -0.8 | **< 0.001** | 0.7 | 0.2, 1.1 | **0.004** | 0.9 | 0.1, 1.7 | **0.028** | 0.4 | 0.1, 0.8 | **0.046** |
| LM (g) | 13762 | 11075, 16448 | **< 0.001** | 4279 | 1593, 6966 | **0.004** | 4405 | 1453, 7357 | **0.010** | 4153 | -336, 8642 | 0.098 |
| FM (g) | -2297 | -3673, -921 | **0.002** | 2101 | 725, 3477 | **0.004** | 2906 | 482, 5330 | **0.028** | 1296 | -8, 2601 | 0.079 |
| FM (%) | -5.9 | -7.7, -4.2 | **< 0.001** | 1.7 | 0.0, 3.5 | 0.058 | 2.6 | -0.5, 5.7 | 0.103 | 0.9 | -0.8, 2.5 | 0.311 |
| BMC (g) | 638 | 456, 821 | **< 0.001** | 411 | 229, 594 | **< 0.001** | 393 | 189, 596 | **0.002** | 430 | 128, 733 | **0.026** |
| BMD (g/cm^2^) | 0.051 | 0.003, 0.099 | **0.042** | 0.131 | 0.082, 0.179 | **< 0.001** | 0.103 | 0.040, 0.166 | **0.005** | 0.159 | 0.085, 0.232 | **0.001** |
| BMD Z-score | -0.95 | -1.42, -0.49 | **< 0.001** | 1.23 | 0.77, 1.70 | **< 0.001** | 0.98 | 0.28, 1.67 | **0.012** | 1.49 | 0.88, 2.10 | **< 0.001** |
| Trunk LM (g) | 5940 | 4667, 7213 | **< 0.001** | 1551 | 278, 2824 | **0.020** | 1313 | 8, 2617 | 0.055 | 1790 | -396, 3977 | 0.121 |
| Trunk FM (g) | -568 | -1280, 144 | 0.116 | 1124 | 412, 1836 | **0.004** | 1322 | 107, 2536 | **0.041** | 927 | 183, 1670 | **0.046** |
| Trunk BMC (g) | 184 | 119, 249 | **< 0.001** | 201 | 135, 266 | **< 0.001** | 180 | 101, 260 | **< 0.001** | 221 | 118, 324 | **0.001** |
| Arms LM (g) | 2347 | 1944, 2750 | **< 0.001** | 891 | 488, 1294 | **< 0.001** | 988 | 543, 1433 | **< 0.001** | 794 | 121, 1466 | **0.046** |
| Arms FM (g) | -269 | -455, -83 | **0.006** | 170 | -16, 356 | 0.072 | 293 | -24, 609 | 0.073 | 47 | -149, 243 | 0.631 |
| Arms BMC (g) | 118 | 88, 148 | **< 0.001** | 57 | 27, 86 | **0.001** | 57 | 22, 92 | **0.005** | 56 | 8, 105 | **0.046** |
| Legs LM (g) | 5032 | 3895, 6170 | **< 0.001** | 1790 | 652, 2927 | **0.004** | 2022 | 607, 3438 | **0.011** | 1557 | -224, 3338 | 0.112 |
| Legs FM (g) | -1675 | -2266, -1085 | **< 0.001** | 891 | 301, 1482 | **0.005** | 1284 | 270, 2297 | **0.022** | 499 | -106, 1104 | 0.121 |
| Legs BMC (g) | 320 | 243, 396 | **< 0.001** | 136 | 60, 213 | **0.002** | 119 | 31, 206 | **0.015** | 154 | 28, 280 | **0.046** |

*Note:* interval BM, body mass; CI; Confidence, tot LM, lean mass; FM, fat mass; FM (%) relative fat mass; BMC, bone mineral content; BMD; bone mineral density, FFMI, fat free mass index; FMI, fat mass index. Data are presented as mean ± *SD.*

Supplementary Table 2. Elite athletics competitors (n=53) off-season to in-season within-group regional and whole-body composition change.

|  | Female | | | | | | Male | | | | | | All athletes | | | | | |
| --- | --- | --- | --- | --- | --- | --- | --- | --- | --- | --- | --- | --- | --- | --- | --- | --- | --- | --- |
|  | Endurance | | | Power | | | Endurance | | | Power | | | Female | | | Male | | |
| Variable | Diff | 95% CI | p-val | Diff | 95% CI | p-val | Diff | 95% CI | p-val | Diff | 95% CI | p-val | Diff | 95% CI | p-val | Diff | 95% CI | p-val |
| BM (kg) | -0.8 | -1.6, 0.0 | 0.235 | 0.1 | -1.0, 1.2 | 0.800 | 0.8 | -0.2, 1.9 | 0.349 | 1.4 | 0.6, 2.3 | **0.003** | -0.3 | -1.0, 0.3 | 0.462 | 1.1 | 0.5, 1.8 | **0.004** |
| FFMI (kg/m^2^) | 0.1 | -0.1, 0.3 | 0.685 | 0.2 | 0.0, 0.4 | **0.041** | 0.3 | 0.1, 0.5 | **0.016** | 0.4 | 0.2, 0.6 | **0.001** | 0.1 | 0.0, 0.3 | 0.061 | 0.4 | 0.2, 0.5 | **< 0.001** |
| FMI (kg/m^2^) | -0.1 | -0.4, 0.2 | 0.685 | -0.2 | -0.4, 0.1 | 0.263 | -0.1 | -0.3, 0.2 | 0.622 | 0.0 | -0.1, 0.2 | 0.823 | -0.1 | -0.3, 0.1 | 0.259 | 0.0 | -0.2, 0.1 | 0.840 |
| LM (g) | 210 | -323, 744 | 0.685 | 618 | 133, 1103 | **0.041** | 1097 | 396, 1797 | **0.016** | 1416 | 703, 2130 | **0.001** | 414 | 54, 775 | 0.061 | 1257 | 757, 1757 | **< 0.001** |
| FM (g) | -334 | -1149, 481 | 0.685 | -500 | -1271, 270 | 0.263 | -240 | -997, 516 | 0.622 | 95 | -357, 547 | 0.823 | -417 | -978, 144 | 0.259 | -73 | -513, 368 | 0.840 |
| FM (%) | -0.3 | -1.6, 1.0 | 0.833 | -0.8 | -1.6, 0.1 | 0.158 | -0.5 | -1.5, 0.5 | 0.622 | -0.1 | -0.7, 0.4 | 0.823 | -0.5 | -1.3, 0.3 | 0.297 | -0.3 | -0.9, 0.3 | 0.502 |
| BMC (g) | 7 | -4, 19 | 0.502 | 22 | 7, 38 | **0.024** | 7 | -16, 29 | 0.622 | 24 | 5, 42 | **0.023** | 15 | 5, 24 | **0.015** | 15 | 1, 30 | **0.075** |
| BMD (g/cm^2^) | 0.022 | 0.001, 0.043 | 0.235 | 0.039 | 0.022, 0.057 | **0.001** | 0.032 | 0.007, 0.058 | 0.054 | 0.036 | 0.020, 0.051 | **0.001** | 0.031 | 0.017, 0.044 | **< 0.001** | 0.034 | 0.019, 0.049 | **< 0.001** |
| BMD Z-score | 0.35 | 0.15, 0.55 | **0.009** | 0.44 | 0.19, 0.68 | **0.008** | 0.46 | 0.13, 0.78 | **0.033** | 0.44 | 0.31, 0.58 | **< 0.001** | 0.39 | 0.23, 0.55 | **< 0.001** | 0.45 | 0.27, 0.63 | **< 0.001** |
| Trunk LM (g) | -43 | -402, 316 | 0.862 | 232 | -88, 552 | 0.234 | 1089 | 578, 1601 | **0.001** | 612 | 227, 996 | **0.007** | 95 | -146, 335 | 0.538 | 851 | 531, 1170 | **< 0.001** |
| Trunk FM (g) | -342 | -803, 119 | 0.403 | -423 | -850, 3 | 0.110 | 55 | -516, 626 | 0.847 | -32 | -333, 269 | 0.884 | -383 | -696, -69 | 0.061 | 12 | -311, 334 | 0.943 |
| Trunk BMC (g) | -8 | -17, 2 | 0.403 | 9 | 0, 17 | 0.110 | 2 | -16, 20 | 0.847 | 0 | -14, 15 | 0.960 | 0 | -6, 7 | 0.903 | 1 | -10, 13 | 0.898 |
| Legs LM (g) | 227 | -72, 525 | 0.403 | 324 | 67, 581 | **0.041** | 95 | -158, 348 | 0.622 | 632 | 227, 1037 | **0.007** | 275 | 79, 472 | **0.030** | 363 | 124, 602 | **0.009** |
| Legs FM (g) | 4 | -282, 291 | 0.976 | -58 | -351, 235 | 0.735 | 59 | -107, 225 | 0.622 | 46 | -120, 213 | 0.818 | -27 | -232, 178 | 0.899 | 52 | -65, 170 | 0.502 |
| Legs BMC (g) | 11 | 6, 17 | **0.001** | 12 | 4, 19 | **0.024** | 11 | 0, 23 | 0.150 | 17 | 9, 26 | **0.001** | 12 | 7, 16 | **< 0.001** | 14 | 7, 21 | **< 0.001** |
| Arms LM (g) | 32 | -66, 130 | 0.725 | 86 | -21, 192 | 0.193 | -93 | -256, 69 | 0.619 | 189 | 44, 334 | **0.023** | 59 | -14, 131 | 0.231 | 48 | -61, 157 | 0.502 |
| Arms FM (g) | -14 | -105, 77 | 0.856 | -37 | -134, 60 | 0.507 | 44 | -56, 143 | 0.622 | 84 | 18, 150 | **0.023** | -26 | -92, 41 | 0.538 | 64 | 4, 123 | 0.075 |
| Arms BMC (g) | -1 | -4, 3 | 0.833 | 1 | -2, 4 | 0.507 | -5 | -14, 5 | 0.622 | 11 | 2, 19 | **0.023** | 0 | -2, 2 | 0.903 | 3 | -3, 9 | 0.502 |

*Note:* BM, body mass; CI, Confidence interval; tot LM, lean mass; FM, fat mass; FM (%) relative fat mass; BMC, bone mineral content; BMD; bone mineral density, FFMI, fat free mass index; FMI, fat mass index. Data are presented as mean ± *SD.*

Supplementary Table 3. Between-group difference in off-season to in-season regional and whole-body composition change in athletics competitors (n=53; F=27, M=26).

|  |  | | |  | | | Female | | | Male | | |
| --- | --- | --- | --- | --- | --- | --- | --- | --- | --- | --- | --- | --- |
|  | Male − Female | | | Power − Endurance | | | Power − Endurance | | | Power − Endurance | | |
| Variable | Diff | 95% CI | p-val | Diff | 95% CI | p-val | Diff | 95% CI | p-val | Diff | 95% CI | p-val |
| BM (kg) | 1.5 | 0.5, 2.4 | **0.027** | 0.8 | -0.2, 1.7 | 0.378 | 0.9 | -0.4, 2.3 | 0.636 | 0.6 | -0.8, 2.0 | 0.728 |
| FFMI (kg/m2) | 0.2 | 0.0, 0.4 | 0.078 | 0.1 | -0.1, 0.3 | 0.564 | 0.1 | -0.1, 0.4 | 0.636 | 0.1 | -0.2, 0.4 | 0.728 |
| FMI (kg/m2) | 0.1 | -0.1, 0.3 | 0.715 | 0.0 | -0.2, 0.2 | 0.923 | -0.1 | -0.4, 0.3 | 0.845 | 0.1 | -0.2, 0.4 | 0.728 |
| LM (g) | 843 | 226, 1459 | **0.048** | 363 | -253, 980 | 0.564 | 407 | -314, 1128 | 0.636 | 320 | -681, 1320 | 0.728 |
| FM (g) | 344 | -369, 1058 | 0.715 | 85 | -628, 798 | 0.923 | -166 | -1287, 955 | 0.845 | 335 | -546, 1217 | 0.728 |
| FM (%) | 0.2 | -0.8, 1.2 | 0.875 | 0.0 | -1.0, 0.9 | 0.923 | -0.5 | -2.0, 1.1 | 0.845 | 0.4 | -0.8, 1.5 | 0.728 |
| BMC (g) | 0 | -17, 18 | 0.967 | 16 | -1, 33 | 0.282 | 15 | -4, 34 | 0.636 | 17 | -12, 46 | 0.728 |
| BMD (g/cm^2^) | 0.003 | -0.017, 0.024 | 0.902 | 0.010 | -0.010, 0.031 | 0.590 | 0.017 | -0.010, 0.045 | 0.636 | 0.003 | -0.027, 0.033 | 0.918 |
| BMD Z-score | .05 | -0.18, 0.29 | 0.860 | 0.04 | -0.20, 0.27 | 0.923 | 0.09 | -0.23, 0.41 | 0.842 | -0.01 | -0.37, 0.34 | 0.935 |
| Trunk LM (g) | 756 | 356, 1156 | **0.007** | -101 | -502, 299 | 0.923 | 275 | -206, 756 | 0.636 | -478 | -1118, 162 | 0.594 |
| Trunk FM (g) | 394 | -56, 844 | 0.240 | -84 | -534, 366 | 0.923 | -82 | -709, 546 | 0.845 | -87 | -733, 558 | 0.918 |
| Trunk BMC (g) | 1 | -12, 14 | 0.967 | 7 | -6, 21 | 0.564 | 16 | 3, 29 | 0.239 | -2 | -24, 21 | 0.918 |
| Legs LM (g) | 88 | -222, 397 | 0.809 | 317 | 8, 627 | 0.254 | 97 | -296, 491 | 0.845 | 537 | 59, 1015 | 0.161 |
| Legs FM (g) | 79 | -157, 316 | 0.778 | -37 | -274, 199 | 0.923 | -62 | -473, 348 | 0.845 | -12 | -247, 223 | 0.918 |
| Legs BMC (g) | 3 | -6, 11 | 0.778 | 3 | -5, 11 | 0.816 | 0 | -9, 9 | 0.968 | 6 | -8, 20 | 0.728 |
| Arms LM (g) | -11 | -142, 120 | 0.967 | 168 | 37, 299 | 0.121 | 53 | -91, 198 | 0.845 | 282 | 64, 500 | 0.161 |
| Arms FM (g) | 89 | 0, 179 | 0.171 | 9 | -81, 98 | 0.923 | -23 | -156, 110 | 0.845 | 40 | -80, 159 | 0.728 |
| Arms BMC (g) | 3 | -4, 10 | 0.776 | 9 | 2, 15 | 0.121 | 2 | -3, 6 | 0.845 | 15 | 3, 28 | 0.161 |

*Note.* BM, body mass; CI, Confidence interval; tot LM, lean mass; FM, fat mass; FM (%) relative fat mass; BMC, bone mineral content; BMD; bone mineral density, FFMI, fat free mass index; FMI, fat mass index. Data are presented as mean ± *SD.*

Supplementary Table 4. Off-season to in-season meaningful (LSC) DXA changes for whole and regional body composition in elite athletics competitors (n=53; F=27, M=26).

|  |  |  |  |  |  |  |  |  |  |  |  |  |  |  |
| --- | --- | --- | --- | --- | --- | --- | --- | --- | --- | --- | --- | --- | --- | --- |
|  | **Female** | | **Male** | |  | **Female** | | **Male** | |  | **Female** | | **Male** | |
| **Change** | **Endurance** | **Power** | **Endurance** | **Power** | **Change** | **Endurance** | **Power** | **Endurance** | **Power** | **Change** | **Endurance** | **Power** | **Endurance** | **Power** |
| **FM whole body** | |  |  |  | **LM whole body** | |  |  |  | **BMC whole body** | |  |  |  |
| Increase | 4 (33%) | 4 (27%) | 3 (33%) | 5 (29%) | Increase | 4 (33%) | 9 (60%) | 7 (78%) | 11 (65%) | Increase | 2 (17%) | 6 (40%) | 1 (11%) | 7 (41%) |
| No Change | 3 (25%) | 5 (33%) | 2 (22%) | 6 (35%) | No Change | 5 (42%) | 5 (33%) | 1 (11%) | 4 (24%) | No Change | 9 (75%) | 9 (60%) | 7 (78%) | 9 (53%) |
| Decrease | 5 (42%) | 6 (40%) | 4 (44%) | 6 (35%) | Decrease | 3 (25%) | 1 (7%) | 1 (11%) | 2 (12%) | Decrease | 1 (8%) | 0 (0%) | 1 (11%) | 1 (6%) |
|  |  |  |  |  |  |  |  |  |  |  |  |  |  |  |
| **FM arms** | |  |  |  | **LM arms** | |  |  |  | **BMC arms** | | |  |  |
| Increase | 2 (17%) | 3 (20%) | 5 (56%) | 8 (47%) | Increase | 2 (17%) | 6 (40%) | 1 (11%) | 8 (47%) | Increase | 0 (0%) | 0 (0%) | 1 (11%) | 6 (35%) |
| No Change | 6 (50%) | 7 (47%) | 2 (22%) | 8 (47%) | No Change | 9 (75%) | 8 (53%) | 5 (56%) | 9 (53%) | No Change | 12 (100%) | 15 (100%) | 6 (67%) | 11 (65%) |
| Decrease | 4 (33%) | 5 (33%) | 2 (22%) | 1 (6%) | Decrease | 1 (8%) | 1 (7%) | 3 (33%) | 0 (0%) | Decrease | 0 (0%) | 0 (0%) | 2 (22%) | 0 (0%) |
|  |  |  |  |  |  |  |  |  |  |  |  |  |  |  |
| **FM trunk** | |  |  |  | **LM trunk** | |  |  |  | **BMC trunk** | |  |  |  |
| Increase | 3 (25%) | 3 (20%) | 4 (44%) | 4 (24%) | Increase | 3 (25%) | 4 (27%) | 6 (67%) | 11 (65%) | Increase | 0 (0%) | 3 (20%) | 1 (11%) | 3 (18%) |
| No Change | 4 (33%) | 5 (33%) | 3 (33%) | 8 (47%) | No Change | 6 (50%) | 9 (60%) | 3 (33%) | 5 (29%) | No Change | 10 (83%) | 11 (73%) | 7 (78%) | 10 (59%) |
| Decrease | 5 (42%) | 7 (47%) | 2 (22%) | 5 (29%) | Decrease | 3 (25%) | 2 (13%) | 0 (0%) | 1 (6%) | Decrease | 2 (17%) | 1 (7%) | 1 (11%) | 4 (24%) |
|  |  |  |  |  |  |  |  |  |  |  |  |  |  |  |
| **FM legs** |  |  |  |  | **LM legs** |  |  |  |  | **BMC legs** | |  |  |  |
| Increase | 4 (33%) | 4 (27%) | 3 (33%) | 6 (35%) | Increase | 5 (42%) | 9 (60%) | 3 (33%) | 10 (59%) | Increase | 4 (33%) | 5 (33%) | 3 (33%) | 8 (47%) |
| No Change | 4 (33%) | 6 (40%) | 5 (56%) | 8 (47%) | No Change | 7 (58%) | 4 (27%) | 5 (56%) | 5 (29%) | No Change | 8 (67%) | 10 (67%) | 6 (67%) | 8 (47%) |
| Decrease | 4 (33%) | 5 (33%) | 1 (11%) | 3 (18%) | Decrease | 0 (0%) | 2 (13%) | 1 (11%) | 2 (12%) | Decrease | 0 (0%) | 0 (0%) | 0 (0%) | 1 (6%) |

*Note:* FM; fat mass, LM; lean mass, BMC, bone mineral content.
